# Trends in prescribing optimal medical therapy at discharge following percutaneous coronary intervention in a tertiary care hospital in the UAE

**DOI:** 10.3389/fcvm.2025.1522216

**Published:** 2025-09-24

**Authors:** Maryam Charehjoo, Seeba Zachariah, Karim Ghannem, Firas Alani, Kimberly McKeirnan

**Affiliations:** ^1^ Department of Pharmacy Practice, College of Pharmacy, Gulf Medical University, Ajman, United Arab Emirates; ^2^ Department of Cardiology, Thumbay University Hospital, Ajman, United Arab Emirates; ^3^ Department of Cardiology, American Hospital, Dubai, United Arab Emirates; ^4^ College of Pharmacy and Pharmaceutical Sciences, Washington State University, Washington, WA, United States

**Keywords:** optimal medical therapy, coronary artery disease, percutaneous coronary intervention, guideline adherence, acute coronary syndrome, coronary reperfusion

## Abstract

**Background:**

Optimal medical therapy (OMT) is recommended in patients with coronary artery disease (CAD) following percutaneous coronary intervention (PCI) to enhance the clinical outcomes and support secondary prevention. However, real-world data on OMT prescription practices in the United Arab Emirates (UAE) remain limited. This study aimed to evaluate the prevalence and determinants of OMT prescription at hospital discharge in a UAE tertiary care setting.

**Methods:**

This retrospective observational study included 103 consecutive patients who underwent PCI between January 2021 and June 2023 at a tertiary academic hospital in the UAE. Demographic and clinical data, including comorbidities and discharge medications, were collected from electronic medical records. OMT was defined as the concurrent prescription of aspirin, a P2Y12 inhibitor, statin, *β*-blocker, and either an angiotensin-converting enzyme inhibitor (ACEI) or angiotensin receptor blocker (ARB). Patients were stratified into OMT and non-OMT groups. Categorical variables were compared using the chi-square test or Fisher's exact test, as appropriate, while non-normally distributed continuous variables were analyzed using the Mann–Whitney *U*-test. Multivariate logistic regression was used to identify factors independently associated with OMT prescription at discharge.

**Results:**

Among the 103 patients, the median age was 49.0 years [interquartile range (IQR): 44.3–54.1], with a predominance of males (93.2%) and non-Arabs (74.8%). OMT was prescribed at discharge in 39 patients (37.9%). Multivariate analysis revealed that diabetes (adjusted odds ratio [aOR] = 3.86, 95% confidence interval [CI]: 1.42–10.52, *p* = 0.01), and hypertension (aOR = 5.99, 95% CI: 2.04–17.60, *p* = 0.001) were significantly associated with higher odds of OMT prescription. In contrast, age >50 years (aOR = 0.23, 95% CI: 0.08–0.65, *p* = 0.01) and the presence of acute heart failure (aOR = 0.06, 95% CI: 0.01–0.60, *p* = 0.02) were associated with lower odds.

**Conclusion:**

The rate of OMT prescriptions at discharge was comparable to international studies, though suboptimal. Diabetes and hypertension were positive predictors, while advanced age, and acute heart failure were negative predictors of OMT prescription. Multicenter studies with larger sample size would be needed to get more details. These findings suggest a need for targeted interventions to improve adherence to guideline-directed therapy. Future multicenter studies with larger sample sizes are warranted to validate these observations.

## 1 Introduction

Cardiovascular diseases continue to be the leading cause of death globally, accounting for over 20.5 million deaths in 2021. Among these, coronary artery disease (CAD) is the most prevalent form, often presenting as acute coronary syndrome (ACS) or stable ischemic heart disease (1). CAD results from the narrowing or blockage of coronary arteries, leading to reduced or absent of blood flow to the heart. Percutaneous coronary intervention (PCI) is a widely utilized revascularization strategy to restore blood flow in CAD patients, particularly in case of ACS. Despite technological advances and procedural improvements, a substantial proportion of patients continue to experience adverse outcomes and mortality in the years following PCI (2).

Long-term prognosis in CAD patients is strongly influenced by adherence to evidence-based pharmacological and non-pharmacological therapies (3). Medications shown to reduce mortality and major cardiovascular events (such as myocardial infarction or stroke) include antiplatelet drugs (aspirin plus P2Y12 inhibitors), beta-blockers, angiotensin-converting enzyme inhibitors(ACEI)/angiotensin receptor blockers(ARBs), and statins. These are recommended as secondary prevention therapy in patients with established CAD, including those who have undergone coronary artery bypass grafting (CABG) or percutaneous coronary intervention (PCI) (4–9). When used together, this combination—commonly referred to as Optimal Medical Therapy (OMT)—has been shown to significantly reduce the risk of recurrent cardiovascular events and improve survival. Dual therapy involving a P2Y12 inhibitor combined with an oral anticoagulant as a substitute for aspirin is also regarded as OMT. Multiple studies have underscored the clinical benefits of OMT when implemented alongside PCI (10–13).

Despite strong guideline recommendations, the presecription of OMT at hospital discharge remains inconsistent and often suboptimal across various healthcare settings. Observed discrepancies in OMT use have been attributed to differences in clinical practice patterns, healthcare system structures, and patient-related factors (12, 14–16). In the United Arab Emirates (UAE), data on OMT prescription practices following PCI are limited. A better understanding of these patterns is essential to identify gaps in care and develop strategies to optimize treatment adherence. This study aims to evaluate the prescribing trends of OMT at discharge following PCI in a tertiary-care hospital in the UAE and to investigate the factors associated with OMT use. These findings may provide valuable insights for enhancing compliance with guideline-directed medical therapy in this regional context.

## 2 Methods

### 2.1 Study populaton and data collection

This retrospective observational cohort study was conducted in a 350 bedded tertiary care academic hospital in the private sector, in the northern Emirate of UAE. List of all patients who underwent PCI at the study site between January 2021 and June 2023 were obtained from the hospital medical records department.

A total of 178 patients were initially identified from hospital medical records. Inclusion criteria was patients aged ≥18 years of either gender who underwent PCI during the study period. Patients with incomplete medical records or those who did not survive till discharge, patients with severe bleeding disorders or severe liver disease or renal impairment, or reported hypersensitivity to any of the OMT drug classess were excluded.

Data were collected from the hospital's electronic medical records through individual chart review capturing baseline patient characteristics, clinical history, procedural data, and home medications. Information was systematically collected at the discharge and during follow-up visits at 1 week, 1 month, 6 months, and 12 months. Key variables included age, gender, nationality, body mass index (BMI), smoking and alcohol use, marital status, comorbidities (such as hypertension and diabetes), previous cardiovascular disease history, number of stents used, and medications prescribed during hospitalization and at discharge along with complications if any reported during the hospitalization period.

The study protocol was approved by the Institutional Review Board (IRB) of Gulf Medical University (Ref. no. IRB-COP-STD-48-NOV-2023) and adhered to the ethical guidelines of the 2013 Declaration of Helsinki. Due to the retrospective nature of the study, informed consent was waived. Patient onfidentiality was maintained by de-identifying data prior to analysis.

### 2.2 Study definitions and outcomes

The primary outcome of this study was to find the poportion of patients prescribed OMT at discharge. Secondary outcomes included assess the temporal trends in OMT prescriptions, effect of patient factors in OMT prescriptions. OMT was defined as the prescription of a combination of aspirin, a P2Y12 inhibitor (such as clopidogrel or ticagrelor), a statin, a beta-blocker, and an ACEI or ARB. Data were also stratified by patients with ACS and those who underwent elective PCI.

### 2.3 Statistical analysis

The Kolmogorov–Smirnov and Shapiro–Wilk test was done to assess the normality of the data set. Continuous variables that did not follow a normal distribution were reported as median and interquartile range (IQR). Comparisons between groups for continuous variables were performed using the Mann–Whitney *U*-test. Categorical variables were presented as counts and percentages and group comparisons were conducted using the the Chi-square test.

To identify the factors associated with OMT prescription at discharge, binary logistic regression was used. Predictor variables included age, gender, comorbidities (hypertension, diabetes, dyslipidemia), smoking status, and previous cardiovascular history. The strength of association between these factors and OMT prescription was expressed as Odds ratios (ORs) with corresponding 95% confidence intervals (CIs). An alpha value of *p* < 0.05 was considered statistically significant. All statistical analyses were performed using IBM SPSS version 29.

## 3 Results

### 3.1 Baseline characteristics

A total of 178 patients underwent coronary angiography. Of these, 72 patients were excluded based on the following criteria: 2 patients died prior to intervention, 4 had normal coronary arteries, 16 were referred for coronary artery bypass grafting (CABG), and 48 were managed conservatively. Additionally, 2 patient records were duplicates. Of the remaining cohort, 106 patients received percutaneous coronary intervention (PCI), among whom 3 did not survive to hospital discharge. Thus, the final study population comprised 103 patients who underwent PCI and were discharged home. All patients were followed throughout hospitalization, up to discharge. A total of 103 patients were included in the analysis, with 64 patients in the non-optimal medical therapy (No OMT) group and 39 in the optimal medical therapy (OMT) group. Tables 1, 2 present the patient charecteristics of the study cohort.

**Table 1:** Patient characteristics.

Variable		Total (*n* = 103) n (%)	No OMT, *n* = 64 n (%)	OMT, *n* = 39 n (%)	*P*-value (Chi-sqare test or ^#^Fischer exact test; *P < 0.05)
Age in years	Age <60 years	91 (88.3)	54 (84.4)	37 (94.9%)	0.11
	Age≥60 years	12 (11.7)	10 (15.6)	2 (5.1)	
Ethnicity	Arab	26 (25.2%)	19 (29.7)	7 (17.9)	0.18
	Non Arab	77 (74.8%)	45 (70.3)	32 (82.1)	
Gender	Male	96 (93.2)	58 (90.6)	38 (97.4)	0.18
	Female	7 (6.8)	6 (9.4)	1 (2.6)	
Smoking	Non smoker	63 (61.2)	37 (57.8)	26 (66.7)	0.37
	Smoker	40 (38.8)	27 (42.2)	13 (33.3)	
Alcoholic	No	97 (94.2)	60 (93.8)	37 (94.9)	0.81
	Yes	6 (5.8)	4 (6.3)	2 (5.1)	
Marital status	Not married	12 (11.7)	8 (12.5)	4 (10.3)	0.73
	Married	91 (88.3)	56 (87.5)	35 (89.7)	
Hypertension	No	40 (38.8)	33 (32.0)	7 (6.8)	<0.001*
	Yes	63 (61.2)	31 (30.1)	32 (31.1)	
Diabetes	No	54 (52.4)	40 (38.8)	14 (13.6)	0.01*
	Yes	49 (47.6)	24 (23.3)	25 (24.3)	
Dyslipidemia	No	55 (53.4%)	36 (35.0)	19 (18.4)	0.46
	Yes	48 (46.6)	28 (27.2)	20 (19.4)	
Ischemic Heart Disease	No	75 (72.8)	47 (45.6)	28 (27.2)	0.86
	Yes	28 (27.2)	17 (16.5)	11 (10.7)	
Stroke/TIA	No	102 (99.0)	63 (61.2)	39 (37.9)	1#
	Yes	1 (1.0)	1 (1.0)	0	
Heart Failure	No	88 (85.4)	57 (55.3)	31 (30.1)	0.18#
	Yes	15 (14.6)	7 (6.8)	8 (7.8)	
Asthma/COPD	No	101 (98.1)	63 (61.2)	38 (36.9)	1#
	Yes	2 (1.9)	1 (1.0)	1 (1.0)	
PCI history	No	13 (12.6)	8 (12.5)	5 (12.8)	0.96
	Yes	90 (87.4)	56 (87.5)	34 (87.2)	
Indication of PCI	Elective	16 9 (15.5)	13 (20.3)	3 (7.7)	0.08
	ACS	87 (84.5)	51 (79.7)	36 (92.3)	
Acute Myocardial infarction	Not MI	25 (24.3)	18 (28.1)	7 (17.9)	0.24
	MI	78 (75.7)	46 (71.9)	32 (82.1)	
C-reactive protein	Normal	70 (68.0)	44 (68.8)	26 (66.7)	0.83
	Elevated	33 (32.0)	20 (31.3)	13 (33.3)	
Neutrophils	Normal	56 (54.9)	39 (61.9)	17 (43.6)	0.07
	Elevated	46 (45.1)	24 (38.1)	22 (56.4)	
White Blood Cells (WBCs)	Normal	41 (39.8)	27 (42.2)	14 (35.9)	0.52
	Elevated	62 (60.2)	37 (57.8)	25 (64.1)	

^#^ Indicates Fisher's Exact Test.

* Indicates *P*-value <0.05.

**Table 2:** Patient characteristics: continuous variables.

Variable	All patients (*n* = 103) Median (IQR)	No OMT, *n* = 64 n (%) Median (IQR)	OMT, *n* = 39 n (%) Median (IQR)	*P*-value (Mann–Whitney U)
Age 1n years	49.0 (44.3–54.1)	49.7 (44.4–55.5)	46.87 (43.6–54.1)	0.92
Weight in Kg	76.0 (71.0–89.0)	76.0 (70.0–89.0)	76.3 (71.0–90.0)	0.25
Height in meter	1.68 (1.6–1.7)	1.69 (1.7–1.7)	1.68 (1.6–1.7)	0.54
BMI in kg/m^2^	27.50 (25.2–31.2)	27.30 (24.9–31.2)	27.50 (25.3–30.9)	0.26
SBP in mmHg	142 (127–159)	140 (125–155)	147 (128–165)	0.44
DBP in mmHg	86 (76–100)	85 (74–95)	88 (77–100)	0.61
Pulse in beats/minute	80 (70–92)	78 (70–90)	82 (66–92)	0.20
Troponin I in ng/ml	1.70 (0.19–14.8)	2.62 (0.3–17.6)	1.38 (0.15–8.5)	0.33
eGFR in ml/min/1.73m^2^	90.00 (79.1–107.6)	91.30 (79.1–107.9)	88.20 (76.4–107.6)	0.92

**Table 3:** Complications during hospitalization.

Complication		Total, *n* (%)	No OMT, *n* (%)	OMT, *n* (%)	*P*-value (Fischer exact test) *P < 0.05
Shock	No	100 (97.1)	61 (59.2)	39 (37.9)	0.17
	Yes	3 (2.9)	3 (2.9)	0	
Acute Heart Failure	No	93 (90.3)	55 (53.4)	38 (36.9)	0.06
	Yes	10 (9.7)	9 (8.7)	1 (1.0)	
Hypotension	No	96 (93.2)	59 (57.3)	37 (35.9)	0.70
	Yes	7 (6.8)	5 (4.9)	2 (1.9)	
Infection	No	97 (94.2)	59 (57.3)	38 (36.9)	0.40
	Yes	6 (5.8)	5 (4.9)	1 (1.0)	
CABG plan	No	99 (96.1)	61 (59.2)	38 (36.9)	1
	Yes	4 (3.9)	3 (2.9)	1 (1.0)	
Arrhythmia	No	89 (86.4)	52 (50.5)	37 (35.9)	0.07
	Yes	14 (13.6)	12 (11.7)	2 (1.9)	

* Indicate *P*-value <0.05.

The majority of patients were under 60 years of age (88.3%), male (93.2%), and non-Arab (74.8%), with no statistically significant differences observed between OMT and No OMT groups for age, ethnicity, or gender. Patients were predominantly of South Asian origin. Most patients were non-smokers (61.2%) and reported no alcohol use (94.2%). Marital status was largely homogenous across groups (88.3% married). Hypertension and diabetes mellitus were significantly more prevalent in the OMT group compared to the No OMT group (*p* < 0.001 and *p* = 0.01, respectively). No significant differences were noted in the prevalence of dyslipidemia, ischemic heart disease, heart failure, or pulmonary comorbidities (asthma/COPD). Prior PCI history and PCI indication (elective vs. ACS) also showed no statistically significant variation between groups, although ACS was more common in the OMT group (92.3%). Inflammatory markers such as C-reactive protein, neutrophil count, and white blood cell levels were elevated in a notable proportion of patients but did not differ significantly between groups.

Table 2 shows that the median age across all patients was 49.0 years (IQR: 44.3–54.1), with no significant difference between groups (*p* = 0.92). Weight, height, and BMI were also comparable between the No OMT and OMT groups (*p*-values: 0.25, 0.54, and 0.26, respectively).

Hemodynamic parameters including systolic blood pressure (SBP), diastolic blood pressure (DBP), and pulse rate showed no statistically significant differences between groups (SBP *p* = 0.44; DBP *p* = 0.61; pulse *p* = 0.20). Troponin I levels were higher in the No OMT group [2.62 ng/ml (IQR: 0.3–17.6)] compared to the OMT group [1.38 ng/ml (IQR: 0.15–8.5)], though the difference was not statistically significant (*p* = 0.33). Estimated glomerular filtration rate (eGFR) was comparable between groups (*p* = 0.92).

Regarding the indication for PCI, 15.5% of patients underwent elective PCI, while 84.5% were treated for acute coronary syndrome (ACS). Among ACS cases, 75.7% had acute myocardial infarction, with STEMI accounting for 56.3%. PCI was performed for the first time in 87.4% of patients. A single drug-eluting stent was used in 45.3% of patients, while two stents were implanted in 33% of cases. Staged PCI was performed in 15.6% of patients. The median hospital stay was 3 days. Follow up visits reported at one week, and at 1, 6, and 12 months were respectively 80%, 59%, 48%, and 37%.

### 3.2 Cardiovascular medications at discharge

Among the 103 patients evaluated at discharge, aspirin (97.1%), P2Y12 inhibitors (99.0%), and statins (99.0%) were the most consistently prescribed agents. Beta blockers were prescribed to 66.0% of patients, while RAAS inhibitors (ACEI/ARB) were used in 57.3%. Notably, SGLT2 inhibitors were prescribed in 39.8% of cases. Prescription rates for Entresto, amiodarone, and calcium channel blockers (CCB) were relatively low (6.8%, 6.8%, and 21.4%, respectively) indicating targeted use in specific comorbid conditions such as heart failure or arrhythmias. Anticoagulant prescriptions were rare at discharge, with warfarin and DOACs prescribed in only 1.0% and 2.9% of patients, respectively. Use of proton pump inhibitors (PPI) was documented in 81.6% of cases.

**Figure 1:**
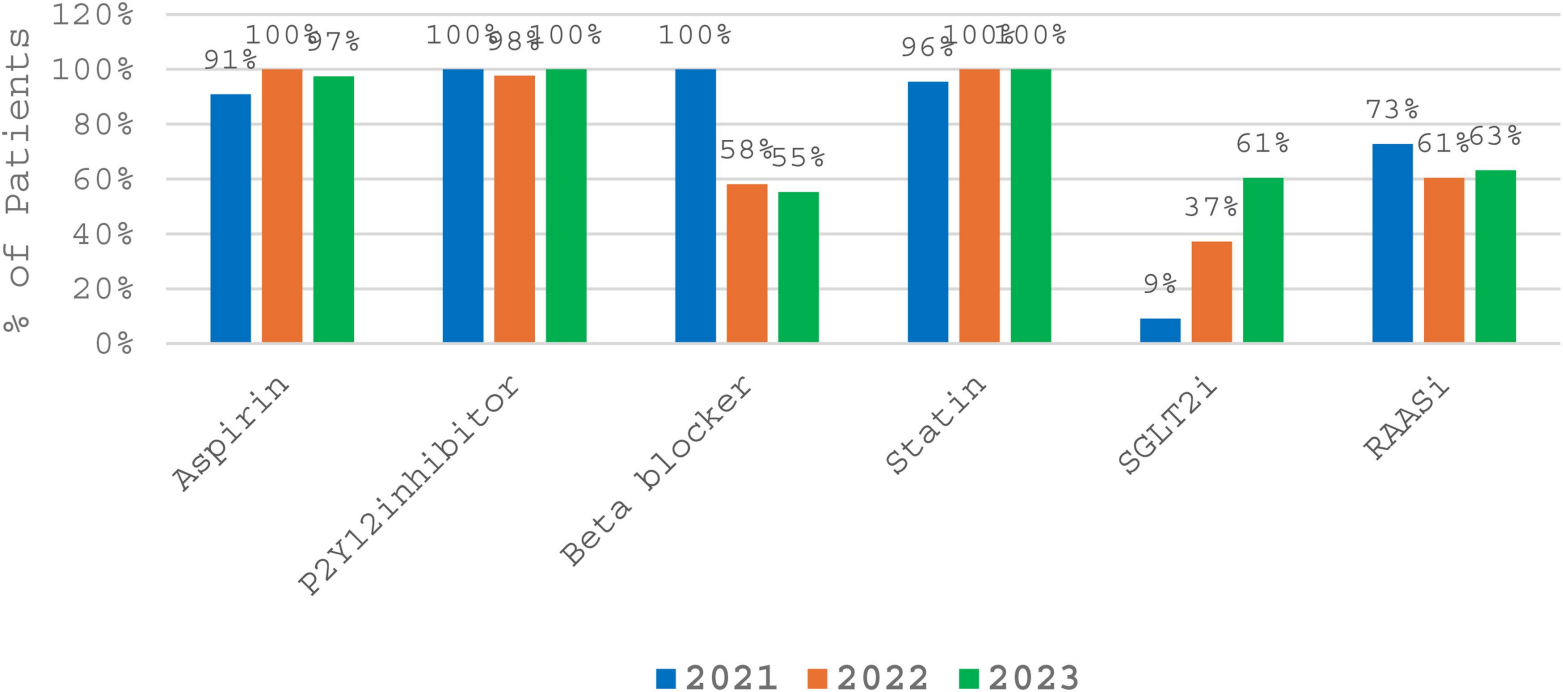
Trends in discharge prescription of cardiovascular medications from 2021 to 2023.

Figure 1 displays the trends in discharge prescription of cardiovascular medications from 2021 to 2023. Over the three-year period, aspirin and P2Y12 inhibitors consistently demonstrated high utilization rates at discharge, maintaining near-universal prescription across all years (aspirin: 91%–100%; P2Y12 inhibitors: 98%–100%). Statin use was also consistently high, reaching 100% in 2022 and 2023. In contrast, the use of beta blockers declined markedly—from 100% in 2021 to 58% and 55% in 2022 and 2023, respectively—suggesting a shift in prescribing patterns or changes in patient eligibility. Use of renin-angiotensin-aldosterone system inhibitors (RAASi) also declined modestly over time (73% in 2021 to 63% in 2023). Notably, prescription of sodium-glucose cotransporter 2 inhibitors (SGLT2i) increased substantially, from 9% in 2021 to 61% in 2023. This reflects the growing integration of SGLT2i into cardiovascular care following expanding indications and stronger evidence supporting their cardioprotective benefits.

**Figure 2:**
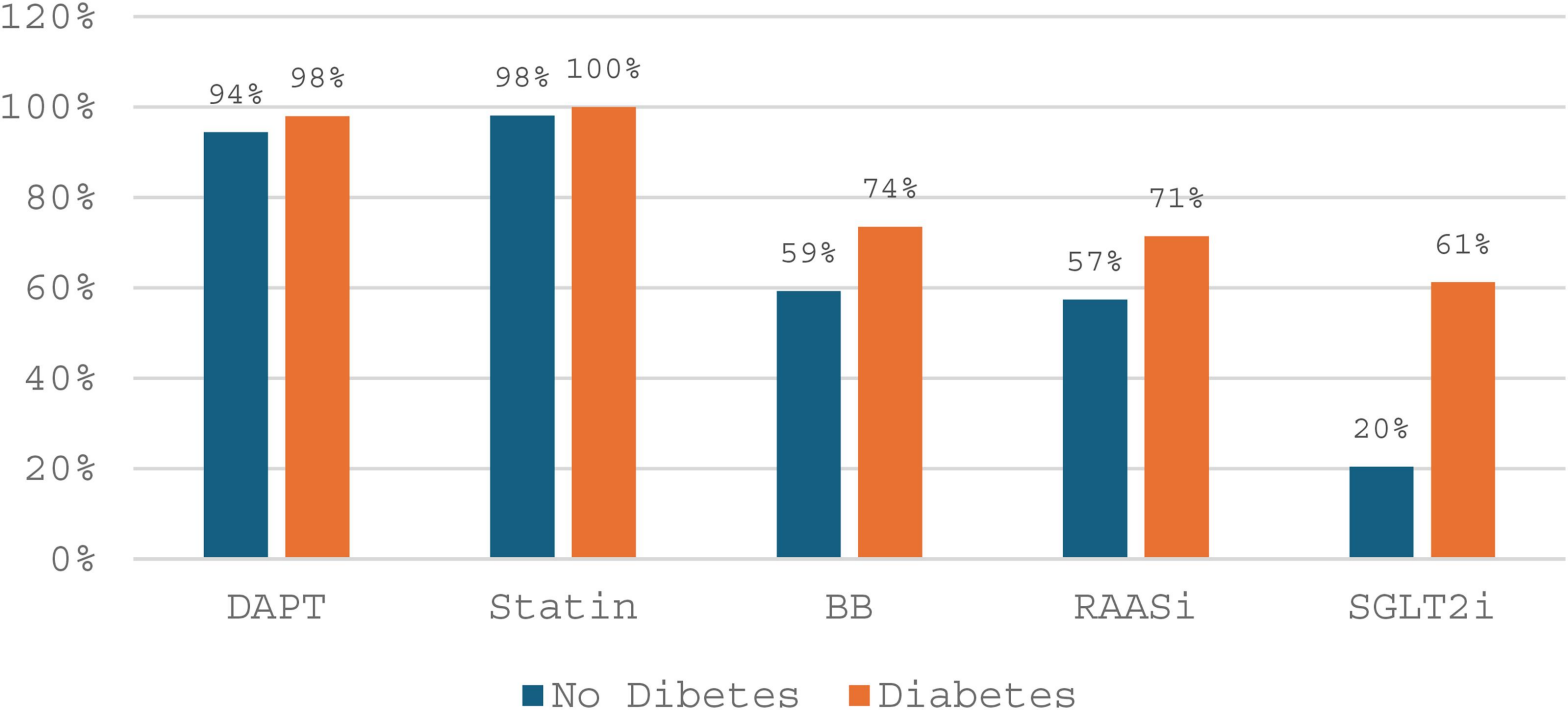
Discharge prescription patterns by diabetes Status.

Figure 2 shows that patients with diabetes had higher rates of cardiovascular medication use at discharge compared to those without diabetes. DAPT and statins were prescribed to nearly all patients in both groups. Beta-blockers, RAAS inhibitors, and especially SGLT2 inhibitors were more frequently used in diabetic patients, reflecting risk-based prescribing patterns.

Table 4 shows that no statistically significant differences were observed in the prescription patterns between elective and ACS groups for the medications assessed. High overall uptake of aspirin, P2Y12 inhibitors, beta blockers, and statins reflects adherence to guideline-directed medical therapy. Slight variations in SGLT2 inhibitor and ACEI/ARB use did not reach statistical significance.

**Table 4:** Comparison of dischcarge prescription of elective and ACS patients.

Discharge medication	Total, *n* = 103 n(%)	Elective, *n* = 16 n(%)	ACS, *n* = 87 n(%)	Chi-square test/Fischer Exact test^#^ *p*-value
Aspirin	100 (97%)	15 (93.8%)	85 (97.7%)	0.40^#^
P2Y12 inhibitor	102 (99%)	15 (93.8%)	87 (100.0%)	0.15^#^
Beta blocker	68 (66%)	12 (75.0%)	56 (64.4%)	0.57^#^
Statin	102 (99%)	16 (100.0%)	86 (98.9%)	1.0^#^
SGLT2 inhibitor	41 (39.8%)	6 (37.5%)	35 (40.2%)	0.84
ACEI/ARB	59 (57.3%)	7 (50.0%)	52 (66.7%)	0.23

^#^ Indicates Fisher's Exact test.

### 3.3 Key predictors of OMT prescription at discharge

We conducted multivariable logistic regression analyses to assess the association between clinical predictors and the likelihood of receiving optimal medical therapy (OMT). Table 5 shows comparison of crude and adjusted odds ratios revealed that while diabetes and hypertension were strong predictors of OMT use in both unadjusted and adjusted models, variables like reduced eGFR, age >50 years, and acute heart failure showed substantially stronger associations after controlling for confounders. Reduced eGFR (<60 ml/min/1.73 m^2^) emerged as a significant independent predictor only after adjustment (aOR 11.4, 95% CI 1.09–119.8, *p* = 0.042).

**Table 5:** Multivariable Logistic Regression Analysis of Predictors of OMT Prescription.

Variables	Crude OR				Model 0 Adjusted OR				Model 1 Adjusted OR				Model 4 Adjusted OR			
Predictor	B	*p*-value	OR	95% CI for OR	B	*p*-value	OR	95% CI for OR	B	*p*-value	OR	95% CI for OR	B	*p*-value	OR	95% CI for OR
eGFR <60	1.25	0.16	3.49	0.607–20.01	2.429	0.05	11.351	1.042–123.69	2.436	0.042	**11**.**42**	1.09–119.84	-	-	-	-
Diabetes	1.091	0.01	**2**.**98**	1.301–6.806	1.304	0.02	**3**.**68**	1.28–10.60	1.327	0.014	**3**.**77**	1.31–10.84	1.35	0.01	**3.86**	1.42–10.52
Hypertension	1.582	0.001	**4**.**86**	1.875–12.627	1.766	0.002	**5**.**85**	1.86–18.36	1.782	0.002	**5**.**94**	1.903–18.56	1.79	0.001	**5.99**	2.04–17.6
Age >50	−0.748	0.08	0.47	0.205–1.094	−1.651	0.01	**0**.**19**	0.06–0.63	−1.717	0.004	**0**.**18**	0.06–0.58	−1.485	0.01	**0.23**	0.08–0.65
Acute HF	−1.827	0.09	0.16	0.020–1.322	−3.035	0.01	**0**.**05**	0.005–0.50	−3.082	0.009	**0**.**05**	0.004–0.47	−2.79	0.02	**0.06**	0.01–0.60
ACS vs. elective	1.12	0.10	3.06	0.812–11.52	0.929	0.26	2.53	0.505–12.71	1.022	0.206	2.79	0.57–13.51	–	–	–	–
Smoking	−0.378	0.37	.685	0.299–1.572	−0.596	0.30	0.551	0.18–1.71	−0.566	0.326	0.57	0.18–1.76	–	–	–	–
Gender	−1.369	0.21	.254	0.029–2.197	−0.69	0.57	0.501	0.05–5.52	–	–	**–**	–	–	–	–	–

Values in bold indicate odds ratios (ORs) with statistical significance (*p*-value <0.05).

Diabetes Mellitus: Diabetes showed a consistent and statistically significant association with OMT prescription across all models. The unadjusted OR was 2.98 (95% CI: 1.30–6.81; *p* = 0.01), which remained significant in both Model 1 (adjusted OR 3.77; 95% CI: 1.31–10.84; *p* = 0.014) and Model 4 (adjusted OR 3.86; 95% CI: 1.42–10.52; *p* = 0.008), reflecting a strong independent association.

Hypertension: Hypertension was also a robust predictor of OMT use, with a crude OR of 4.86 (95% CI: 1.88–12.63; *p* = 0.001). The association remained significant in both Model 1 (adjusted OR 5.94; 95% CI: 1.90–18.56; *p* = 0.002) and Model 4 (adjusted OR 5.99; 95% CI: 2.04–17.60; *p* = 0.001), reinforcing its role as a key determinant in prescribing decisions.

Age >50 Years: Older age was inversely associated with OMT use. Although the crude OR was not statistically significant (OR 0.47; 95% CI: 0.21–1.09; *p* = 0.08), the association became highly significant after adjustment in Model 1 (adjusted OR 0.18; 95% CI: 0.06–0.58; *p* = 0.004) and Model 4 (adjusted OR 0.23; 95% CI: 0.08–0.65; *p* = 0.006), suggesting a lower likelihood of OMT among older patients.

Acute Heart Failure: Acute HF showed a non-significant protective trend in the crude model (OR 0.16; 95% CI: 0.02–1.32; *p* = 0.09), but this association became significant in adjusted analyses. In Model 1, the adjusted OR was 0.05 (95% CI: 0.004–0.47; *p* = 0.009), and in Model 4 it remained consistent (adjusted OR 0.06; 95% CI: 0.006–0.60; *p* = 0.02), indicating markedly lower odds of OMT prescription among patients with acute HF.

ACS vs. Elective PCI: Presentation with acute coronary syndrome (ACS) showed a trend toward higher OMT prescription (crude OR 3.06; 95% CI: 0.81–11.52; *p* = 0.10), but this did not reach significance in adjusted models (Model 1 adjusted OR 2.79; 95% CI: 0.57–13.51; *p* = 0.206), suggesting the association may be influenced by confounding.

eGFR <60 ml/min/1.73 m^2^: In the unadjusted analysis, reduced eGFR was associated with a non-significant increase in odds of receiving OMT (OR 3.49; 95% CI: 0.61–20.01; *p* = 0.16). However, after adjusting for covariates in Model 1, the association became statistically significant (adjusted OR 11.42; 95% CI: 1.09–119.84; *p* = 0.042), indicating that patients with renal impairment were substantially more likely to receive OMT when accounting for comorbidities.

Smoking and Gender: Smoking status and gender were not significantly associated with OMT prescription in either unadjusted or adjusted models. In Model 1, smoking had an adjusted OR of 0.57 (95% CI: 0.18–1.76; *p* = 0.326), and gender had an OR of 0.25 (95% CI: 0.03–2.20; *p* = 0.21).

Diabetes, hypertension, and reduced eGFR emerged as strong positive predictors of OMT use, while age above 50 years and acute HF were associated with lower odds of receiving therapy. These findings suggest targeted interventions may be needed to address underutilization of OMT in older adults and those with acute HF, while CKD may paradoxically act as a trigger for more aggressive treatment due to perceived cardiovascular risk.

### 3.4 OMT prescriptions during follow-up visits

Follow up visits reported at one week, and at 1, 6, and 12 months were respectively 80%, 59%, 48%, and 37%. At discharge, prescription rates were highest for P2Y12 inhibitors (99%), aspirin (97%) and statins (99%). By 12 months, these rates were 94.7%, 89.5% and 100% respectively. Among P2Y12 inhibitors, 78% of patients received ticagrelor 90 mg twice daily, while 20% were prescribed clopidogrel 75 mg once daily. Dual antiplatelet therapy (DAPT) with aspirin and P2Y12 inhibitors were prescribed to 97% of patients at discharge, decreasing to 86.8% at the 12 months follow up. This could be due to the discontinuation of either aspirin or P2Y12 inhibitors at some point. High-intensity statin was prescribed in 89% of cases at discharge.

Complared to antiplatelets and statins, beta-blockers and ACEIs/ARBs had lower prescription rates at discharge (61.1% and 57.3% respectively). Beta-blocker use increased to 78.9% at the 12 month mark, while ACEIs/ARBs use remained relatively stable.

## 4 Discussion

This study examined real-world patterns of medication use following percutaneous coronary intervention (PCI), with a focus on the prescription of optimal medical therapy (OMT) at discharge. OMT was prescribed at discharge in 37.9% of patients. While antiplatelet and statin therapies showed high uptake, the use of beta-blockers, RAAS inhibitors, and SGLT2 inhibitors remained low. Notably, regression analyses identified hypertension, diabetes, reduced renal function, younger age, and absence of acute heart failure as independent predictors of OMT.

### 4.1 Rate of OMT prescription at discharge

The rate of OMT prescription at discharge in our cohort (37.9%) aligns with international findings, ranging from 25% to 60% depending on region and registry (12, 16–21). These variations reflect differences in patient profiles, year of data collection, healthcare infrastructure, insurance coverage, and the differences in operational definition of OMT across studies. Compared to other studies, our cohort consisted of a significantly younger male population.

In our study, aspirin (97.1%), P2Y12 inhibitors (99.0%), and statins (99.0%) prescriptions were higher where as the use of ACEI/ARBs (57.3%) and beta blocker (66%) were lower compared to a large prospective study from New Zealand and Australia (20). Temporal pattern showed increase in the use of SGLT2 inhibitors from 2021 to 2023 while ACEI/ARBs and beta blocker use were decreasing. This change could be due to the guideline updates and more publications on SGLT2 inhibitors. The 2020 European Society of Cardiology guidelines and the 2025 ACC/AHA guidelines do not recommend the use of ACEI/ARBs for all ACS patients unless reduced left ventricular ejection fraction of less than 40%, diabetes or chronic kidney disease or hypertension are present. Beta blockers are recommended during ACS hospitalization unless contraindicated. In case of stable ishcemic heart disease patient with elective PCI, chronic beta blocker use has no benefit in reducing cardiovascular events unless there is other indications like angina, arrhythmia, hypertension, or reduced ejection fraction (3, 22, 23). Clinical inertia, patient comorbidities, or contraindications such as low BP could be the reason for lower use of these drugs. The inclusion of elective PCI cases in our cohort also partly explain the lower overall rate of OMT at discharge.

### 4.2 Predictors of OMT prescription

Multivariable regression analysis highlighted diabetes mellitus and hypertension as robust, independent predictors of OMT prescription. This reflects clinician recognition of elevated cardiovascular risk and adherence to guideline-based risk stratification. Interestingly, renal impairment (eGFR <60) was also associated with greater odds of OMT use after adjustment. This is due to the perceived cardiovascular risk in chronic kidney disease as well as the nephrology consultation that resulted in adding ACEI/ARBs to reduce the proteinuria and CKD progression. Patients above 50 years and those with acute heart failure were significantly less likely to receive OMT. These associations reflect concerns about hypotension, drug tolerability, or polypharmacy in older and acutely unwell individuals. Hypotension and acute heart failure could negatively affect the initiation of ACEI/ARBs as well as beta blockers. Gender and smoking status, commonly studied determinants, did not significantly affect prescribing decisions in our cohort. This observation is consistent with global trends where OMT prescriptions decrease with age despite evidence that OMT provides significant survival benefits across all age groups (24, 25). These results emphasize the need for personalized treatment strategies, especially in the older population, taking into account their frailty and coexisting medical conditions.

### 4.3 Medication trends over time

Longitudinal analysis from 2021 to 2023 revealed stable and high prescription rates of aspirin, P2Y12 inhibitors, and statins, with over 90% of patients consistently receiving these agents. However, β-blocker use declined substantially from 100% in 2021 to 55% in 2023. RAAS inhibitor use also showed a modest decline. These trends reflect a shift in clinical practice, changing patient profiles, or increased recognition of contraindications. Conversely, prescription of SGLT2i rose markedly, from 9% in 2021 to 61% in 2023. This trend mirrors recent international guidelines and trials, supporting the integration of SGLT2i into cardiovascular care.

### 4.4 Clinical implications

Eventhough there were good guideline adherence to antithrombotics and lipid lowering therapy, there are areas of improvement. The observed discrepancies in medication use patterns, especially among older adults and those with heart failure, suggest the need for targeted interventions. Physicians reluctancy to prescribe aggressive medication therapies to avoid adverse effects, not aware of the indication and clincial benefit of the drugs could be the reason for underutilization (26, 27). These may include standardized discharge checklists, involvement of clinical pharmacists, or use of clinical decision-support systems to flag eligible patients not receiving OMT (18–21, 26).According to various studies, prescribing OMT at discharge is linked to better patient adherence, improved clinical outcomes in the long term, and a reduction in 1-year all-cause mortality and major adverse cardiovascular events (MACE) (18–21, 28). In a single-center, real-world observational study involving over 9,000 patients with acute coronary syndrome found that the prescription of OMT at discharge was associated with a reduction in 1-year mortality. Additionally, patients who received OMT without either a beta-blocker or ACEI/ARBs experienced better clinical outcomes compared to those who did not receive OMT (15). Delaying the in-hospital initiation of OMT has consistently been linked to patients either never starting the therapy or experiencing significant delays before it begins (29). With growing PCI volumes in the UAE and similar healthcare systems, ensuring consistent OMT prescription at discharge could yield substantial public health benefits.

### 4.5 Study limitations

Limitations include the single-center design, modest sample size, and observational nature, which limit generalizability. Eventhough the follow up was for 12 months, high attrition rate limited analysis of the follow up data. Medication adherence was not measured in this study.

LVEF is an important factor influencing the prescription of OMT, but was not available for all patients and hence was not analysed. Patients with contraindications to antithrombotic and lipid lowering therapy were not included in the population.

## 5 Conclusion

The proportion of patients discharged with OMT was found to be consistent with figures reported in international literature; however, despite this alignment, the overall prescription rate remained suboptimal relative to ideal standards of care. This suggests that, while prescribing practices may reflect global trends, they still fall short of ensuring universal adherence to guideline-directed therapy at discharge. Among the clinical factors evaluated, the presence of diabetes mellitus and hypertension emerged as positive predictors of OMT utilization. Patients with these comorbidities were more likely to receive appropriate pharmacologic management, likely reflecting clinicians' heightened awareness of cardiovascular risk modification in these populations. Conversely, advanced patient age and a diagnosis of acute heart failure were identified as negative predictors**,** indicating a lower likelihood of OMT initiation or continuation in these subgroups.

## Data Availability

The raw data supporting the conclusions of this article will be made available by the authors, without undue reservation.
